# Restoration and content analysis of ancient manuscripts via color space based segmentation

**DOI:** 10.1371/journal.pone.0282142

**Published:** 2023-03-22

**Authors:** Muhammad Hanif, Anna Tonazzini, Syed Fawad Hussain, Akhtar Khalil, Usman Habib

**Affiliations:** 1 Faculty of Computer Science and Engineering, GIK Institute, Topi, Pakistan; 2 Istituto di Scienza e Tecnologie dell’Informazione “Alessandro Faedo” Area della Ricerca CNR di Pisa, Pisa, Italy; 3 School of computer science, University of Birmingham, Birmingham, United Kingdom; 4 Research and Innovation, iFahja Limited, United Kingdom; 5 Department of AI &; DS, School of Computing, National University of Computer and Emerging Sciences, Islamabad, Pakistan; Universidad de Guadalajara, MEXICO

## Abstract

Ancient manuscripts are a rich source of history and civilization. Unfortunately, these documents are often affected by different age and storage related degradation which impinge on their readability and information contents. In this paper, we propose a document restoration method that removes the unwanted interfering degradation patterns from color ancient manuscripts. We exploit different color spaces to highlight the spectral differences in various layers of information usually present in these documents. At each image pixel, the spectral representations of all color spaces are stacked to form a feature vector. PCA is applied to the whole data cube to eliminate correlation of the color planes and enhance separation among the patterns. The reduced data cube, along with the pixel spatial information, is used to perform a pixel based segmentation, where each cluster represents a class of pixels that share similar color properties in the decorrelated color spaces. The interfering, unwanted classes can thus be removed by inpainting their pixels with the background texture. Assuming Gaussian distributions for the various classes, a Gaussian Mixture Model (GMM) is estimated through the Expectation Maximization (EM) algorithm from the data, and then used to find appropriate labels for each pixel. In order to preserve the original appearance of the document and reproduce the background texture, the detected degraded pixels are replaced based on Gaussian conditional simulation, according to the surrounding context. Experiments are shown on manuscripts affected by different kinds of degradations, including manuscripts from the DIBCO 2018 and 2019 publicaly available dataset. We observe that the use of a few PCA dominant components accelerates the clustering process and provides a more accurate segmentation.

## Introduction

Ancient, historical manuscripts consist of a number of different patterns, or layers of information. Besides the main text and the paper texture, they may also contain other informative features, such as annotations, miniatures, stamps, or non-informative interference’s due to damages caused by storage or other manhandling with the passage of time. Mostly, these damages appear as spots of humidity and molds, or ink seeped from the reverse side, impairing the main text.

An important aim of digital image processing techniques applied to the available digital copies of ancient manuscripts is to provide the scholars with versions that can help them in reading and interpretation of the main contents. Therefore, the main theme of virtual restoration is to put back the manuscripts to their original appearance, by eliminating only the degradation without destroying the other informative features. In this sense, the plurality of manuscript content should be analyzed and discriminated, in such a way to preserve or even highlight the useful patterns and remove the extra useless patterns that can disturb or even make impossible the interpretation or reading of document contents.

Another goal of digital manuscript restoration is to prepare them for computer based automatic word spotting and/or character recognition. In this case, binarization is usually performed as a first step to extract the foreground text against all other features considered as complex background or noise. A wide interest exists about degraded document binarization [1], and a variety of methods have been proposed. Among those, local and adaptive thresholding, or recurrent, convolutional or deep neural networks have been shown to cope, to some extent, with degradations such as uneven illumination, image contrast variation, changes in stroke width and connection, faded ink of faint characters [2–5]. To improve binarization, noise filtering and contrast enhancement are also often applied as a pre-processing step [6].

Thus, in general, binarization is also seen as a way to restore degraded documents. Such an approach is however far away from the concept of virtual restoration, where the main focus is restore the aesthetic look of the document.

The bleed-through distortion is perhaps an exception in this panorama, as its removal has raised a certain interest individually and outside the binarization context. Indeed, strong bleed-through that often occur in historical manuscripts cannot be normally removed by binarization alone. They mainly fail due to the significant overlap of bleed-through with the foreground text and the wide variation of its extent and intensity. Methods specifically designed for bleed-through reduction have been proposed, e.g., in [7], where a recursive unsupervised segmentation is suggested; and in [8], where a conditional random field (CRF) using intensity distribution as prior is presented.

A peculiarity of the bleed-through removal problem is that extra information can be exploited, for example, the verso side information of the manuscript. This allows for the design of algorithms that are able to selectively remove the unwanted interference alone, leaving the rest of the manuscript content unaltered. Examples of such techniques can be found in [9], where a classification is performed by segmenting the recto-verso joint histogram with the aid of ground truths. Also, in [10], where a dual-layer Markov Random Field (MRF) prior is combined with a data term derived from user-labeled pixels, and in [11], where correlated component analysis is used to separate the information layers. In [12], the fidelity of the restored manuscript to the original one is further improved by inpainting the bleed-through pixels, detected by the model-based method in [13], through sparse image representation and dictionary learning.

The performance of the recto-verso based methods depends, however, on the alignment of the two images. Accurate registration is difficult to achieve in this specific case due to document skews, different image resolutions, or wrapped pages when scanning books. Thus, dedicated recto-verso registration algorithms have to be designed. Some joint registration and restoration methods have been proposed in [14–17].

Although the problem of removing severe bleed-through while preserving the useful content is not yet fully resolved, some of the methods mentioned above can be used for virtual restoration of complex manuscripts.

In this paper, we extend the concept of virtual restoration of a manuscript to the removal of degradation patterns of any type, such as spots, bleed-through, non-uniform illuminations, etc. The proposed method uses single-sided RGB manuscripts, thus avoiding the need for recto-verso alignment, and adopt the approach of performing an analysis of their content, that is of individually detecting and locating their constituent patterns. Virtual restoration is then obtained by inpainting the undesired interfering content with the background texture. It is straightforward to see that such an approach can also serve to facilitate the execution of other tasks besides virtual restoration, for instance, document binarization or text extraction, and geometrical and logical page layout analysis.

The remainder of the paper is organized as follows. the next section briefly presents image segmentation using color information and GMM for data clustering. Then, we present the proposed color based segmentation method and discuss the Gaussian texture inpainting in the next section. A set of experimental results and their qualitative evaluation is presented to validate our claim. Concluding remarks are given in the last section.

## Background

In ancient manuscripts acquired in RGB modality, there is strong spectral correlation in patterns associated to same components and high color contrast among different components. This correlation and contrast in spectral information made them perceptually well distinguishable. In fact, some distinctive components, such as initials and stamps, have originally colored in such a way that contrast with the main text or writings. Moreover, the spectral or color homogeneity in components (i.e., text, background, spots, bleed-through, graphical elements etc.) is a distinctive feature of manuscripts with respect to natural scenes. Hence, it is reasonable to assume that clusters of pixels having similar spectral responses might correspond to the same components, and that a color clustering technique can be able to segment the manuscript in its constituent components.

### Color space based segmentation

Color space based segmentation is a critical problem in image analysis and computer vision, with a wide variety of applications. The clustering in these applications depends on the used color space and the homogeneity criterion is based on features derived from the spectral components. These color features, derived from different color spaces, contain significant amount of discriminative information. By definition, a color space is a tool to visualize, create, and specify the color [18]. The choice of the color space for image segmentation is delicate: several color spaces, such as RGB, HSI, and CIELuv have been used, but none of them is optimally suited in the same way for all kinds of images and applications [19].

The most commonly used color space is the RGB color space. Other color spaces are usually derived from the RGB color space by means of either linear or nonlinear transformations [20]. However, several studies have shown that the RGB representation is not optimal for segmentation purposes. Indeed, it has some limitations in conveying the information, since its R, G and B channels are highly correlated, and it fails to mimic the color perception of the human visual system, where the color is better represented in terms of hue, saturation, and intensity [20]. The HSI space, obtained from RGB, overcomes this problem, but both RGB and HSI are not perceptually uniform, meaning that the difference in color perceived by the human eye is not represented by similar distance in those spaces. The CIE color spaces (CIELuv, CIELab) introduce a uniform metric for assessing perceptual differences among colors using the Euclidean distance.

The performances of different color spaces for segmentation purposes have been widely compared in the literature, concluding that no single color space performs in the same way for all kinds of images or segmentation methods [19]. In [21] the CMY color space is suggested to perform at best for segmentation based on clustering. A comparison of ten color spaces for skin detection and segmentation is presented in [22], concluding that the HSV color space produces the best results. Similarly, in [23], the HSV color space is recommended for crop image segmentation. Based on spectral color analysis, in [20] the color space is selected according to the task at hand. A color quantization clustering method, using eigenvalues of the color covariance matrix, is suggested in [24]. Recently, different combinations of color spaces have been evaluated in [25] for segmentation tasks using different databases. They concluded that a combination of different color spaces can be used to improve segmentation results and certain classes of objects are better represented by different color spaces.

### Gaussian mixture model

In the last decade, mixture models using probability density functions (pdf) of pixel attributes emerged as a powerful tool for data clustering [26]. Assuming that each cluster is derived by a single pdf, these methods automatically provide data clusters on the basis of the mixture components that generate them [27]. In particular, Gaussian mixture models (GMM) are very flexible in capturing the distribution of different feature spaces, and have been specifically suggested for color image segmentation [28]. Furthermore, the mixture parameters with Gaussian components can be efficiently estimated through maximum likelihood estimation using the well established expectation maximization (EM) algorithm. For better results, these methods may also incorporate pixel location information to account for spatial smoothness, with the assumption that nearby pixels belong to the same cluster [29]. In mixture models, image segmentation is framed as a hidden variable problem, where the hidden class labels determine which component generates a specific image pixel.

### Our contribution

Based on the discussion above, in this paper we propose to perform color image segmentation through GMMs in conjunction with multiple color space representations, to analyze the content of ancient degraded manuscripts. Unlike the binary restoration, the main focus is to restore the aesthetic look of the manuscript, which has its own importance in processing ancient documents. We thus combine three different color spaces information to create a feature space that is able to capture all the necessary information to discriminate the classes (foreground text, background medium, and a number of other different information/degradation patterns), by highlighting the differences, even slight, in their spectral responses. More specifically, we associate each pixel with its representations in the RGB, CIELUV and CIELAB color spaces, along with its spatial location in the image. The spatial smoothness constraint enforced by pixel spatial information is particularly suited to describe the homogeneity of color usually observed in typical manuscript patterns mentioned above.

In case of our feature space, the color vector coordinates can be considered as a realization of a multivariate Gaussian law and the color image as a realization of a random field, with each random variable described by a GMM. A GMM based clustering can be used for pixel based classification. In order to improve and speed up GMM clustering, we perform principal component analysis (PCA) of the initial data space, to decorrelate it and to reduce its dimension without loosing information. In [30], it has been shown that PCA is particularly beneficial for K-means clustering by eliminating associations between data and improves the quality of segmentation. The PCA data reduction allows to speed up the training process of the GMM model, with a consequent significant reduction of the computational time. In our specific case, we have experimented that a number of 3 dominant PCA components are sufficient to capture the essential information and organize it in a more coherent way. The GMM is estimated through the EM algorithm based on the reduced feature space. Each pixel is labeled as belonging to the class associated to the Gaussian component that returns the highest probability for the feature vector of that pixel.

After segmentation, a virtually restored image of the manuscript with all its informative content is generated by selectively replacing the detected degradation pixels with with an appropriate fill-in pixels that reproduce the textured background. The inpainting is performed via Gaussian conditional simulation [31], accounting for the surrounding context to preserve the aesthetic look of the manuscript.

Experimental results show that the proposed method can be satisfactory used to remove the interference commonly found in ancient manuscripts, and to extract typical salient features. We also test the method also on some images from the DIBCO 2018 data set [32].

## The proposed method

Each pixel *i* in a color image can be defined by a vector $D_{i}={\left[d_{1},d_{2},d_{3},\ldots ,d_{N}\right]}_{i}^{T}$. The dimension *N* depends on the color space used, e.g., *N* = 3, **D**_*i*_ = [*R*, *G*, *B*], for the RGB color space, or *N* > >3 in multispectral or hyperspectral images. For an N-color image with *m* rows and *n* columns there will be *M* = *m* × *n* such vectors, or equivalently *N* spectral planes of size *m* × *n*. The mean vector of all image vectors can be calculated as:

$$\begin{array}{c} \bar{D}=\frac{1}{M}\sum_{i=1}^{M}D_{i} \end{array}$$

The covariance matrix of **D** can be approximated as

$$\begin{array}{c} Cov\left(D\right)=\frac{1}{M}\sum_{i=1}^{M}\left(D_{i}-\bar{D}\right){\left(D_{i}-\bar{D}\right)}^{T} \end{array}$$

To extract the decorrelated spectral bands, one can use PCA. The PCA is based on the eigenvalue decomposition of the covariance matrix, given as:

$$\begin{array}{c} Cov\left(D\right)=V\Delta V^{T} \end{array}$$

where Δ = *diag*(λ_1_, λ_2_…λ_*N*_) is the diagonal matrix composed of the eigenvalues λ_1_, λ_2_…λ_*N*_ of *Cov*(**D**), and **V** is the orthonormal matrix composed of the corresponding *N* dimension eigenvectors of *Cov*(**D**).

The linear transformation defined by **y**_*i*_ = **V**^*T*^**D**_*i*_ (*i* = 1, 2, …, *M*) is the PCA pixel vector, and all these pixel vectors form the PCA bands of the input image. Let the eigenvalues and eigenvectors be arranged in descending order so that λ_1_ > λ_2_ > … > λ_*N*_, thus the first *K* (usually *K* ≪*N*) rows of the matrix **V**^*T*^, namely the first *K* eigenvectors, can be used to find an approximation of the input image. Such PCA bands have the highest contrast or variance in the first band and the lowest contrast or variance in the last band. Therefore, the first *K* PCA bands contain the majority of information residing in the input images and can be used for more effective and accurate analyses because the number of image bands and the amount of image noise involved are reduced.

In the proposed method, we first transform the original *R*, *G* and *B* features of the color manuscript image *I* into a hybrid color space, including RGB, CIELab and CIELuv. In the color feature set, we retain only one *L* channel, since it is same in the CIELab and CIELuv color spaces, thus obtaining the eight distinct color features *R*, *G*, *B*, *L*, *U*, *V*, *A* and *B*. All the color bands are stacked together to form a multispectral image cube *χ*. Each pixel in this cube is a feature vector containing eight color values, from three color spaces. Let *χ*_*i*_ = (*d*_*R*_, *d*_*G*_, *d*_*B*_, *d*_*L*_, *d*_*U*_, *d*_*V*_, *d*_*A*_, *d*_*B*_) be the *i*^*th*^ pixel vector in our data cube, where *d*_*F*_ represents the scalar value observed in the *F* color space. The constructed feature cube is used to find the dominant PCA bands. These PCA bands are mutually orthogonal and their covariance matrix takes the form

$$\begin{array}{c} Cov\left(\chi \right)=diag\left(\lambda_{1},\lambda_{2},\ldots .,\lambda_{K}\right) \end{array}$$

The PCA transformation of the original feature cube *χ* remove the correlation among the bands and facilitate the classification by identifying the optimum linear combination of the spectral bands accounting for the variation of pixel values. We select the first 3 principal components (PC) and pass these PCs along with the pixel spatial location information to the pixel classification step, as a feature space *S*.

### Pixel segmentation

Consider the extracted PCA image **I**_*K*_, *K* = 1, 2, 3 as an unlabeled random sample, with *P* pixels, **I** = (*i*_1_, *i*_2_, …, *I_p_*), drawn from an independent and identical distribution (i.i.d). In this paper, unlabeled sample means that for any pixel *i*_*p*_ ∈ **I**_*K*_ the true sub-population to which *i*_*p*_ belongs is not known. Our main goal is to make accurate statistical inferences on properties of the sub-populations by using only the information of the unlabeled observed image **I**_*K*_.

In general, the classification problem consists of finding *C* classes *C*_1_, *C*_2_, …, *C*_*L*_ of pixels whose feature vectors satisfy a given similarity criterion, such that every *i*_*p*_, *p* = 1, 2, .., *P*, belongs to one of these classes and no *i*_*p*_ belongs to two classes at the same time, that is ${⋃}_{n=1}^{N}C_{n}=I$ and *C*_*l*_ ⋂ *C*_*j*_ = ∅ ∀ *l* ≠ *j*.

One popular way to perform this classification is to model the feature space *S* as a realization of a random field, each random variable described by a Gaussian Mixture Model (GMM) [33]. More precisely, each *i*_*p*_ ∈ *S* is supposed to be drawn from a Gaussian mixture of multivariate normal distributions given as

$$\begin{array}{c} p\left(s|\Theta \right)=\sum_{j=1}^{L}\alpha_{j}G_{j}\left(s|\theta_{j}\right) \end{array}$$ (1)

where Θ = {(*α*_*j*_, *θ*_*j*_)}_*j* = 1,2, …, *L*_, $G_{j}\left(i|\theta_{j}\right)$ is a multivariate Gaussian probability density function with parameters *θ*_*j*_ = (*μ*_*j*_, Λ_*j*_) representing the mean vector and the co-variance matrix, respectively, and *α*_*j*_∈ is the mixing weights, satisfying $\sum_{j=1}^{L}\alpha_{j}=1$.

Assuming that each image pixel is actually drawn from one of the *L* Gaussian components in the mixture of Eq (1), then the classification problem can be reformulated as that of finding the parameters of the *L* Gaussian densities of Eq (1) that best describe *L* homogeneous partitions of the pixels. In other words, for each i.i.d observation *i*_*p*_, generated according to Eq (1), we assume a hidden class label *c*_*l*_, *l* ∈ [1, 2, …, *L*], indicating the density distribution in the mixture that generates it, and estimate as the maximizer of the following distribution

$$\begin{array}{c} p\left(c_{j}|i_{p}\right)=\frac{p\left(c_{j}\right)p\left(i_{p}|c_{j}\right)}{p(i_{p})}\;j=1,2,\ldots ,L \end{array}$$ (2)

given by the Bayes rule and representing the posterior probability that *i*_*p*_ belongs to class *C*_*j*_.

To estimate the unknown parameter set Θ, i.e. *θ*_*j*_ = (*μ*_*j*_, Λ_*j*_) and class probability *p*(*c*_*j*_) = *α*_*j*_, *j* = 1, 2, …, *L*, associated with each cluster, along with probabilities (2) for each observation, we use the EM algorithm. The strength of EM algorithms is based on the concept of incomplete data and complete data, which allows to simplify parameter estimation through Maximum Likelihood. In our setting, the feature space *S* is considered as incomplete data, whereas (*S*, *p*(*c*_*j*_|*i*_*p*_), *j* = 1, 2, …, *L*, *p* = 1, 2, …, *P*) represents the associated complete data. The likelihood of the observed incomplete data is marginal over the hidden class labels of the joint distribution of the complete data.

EM is an iterative algorithm that at each iteration alternates between two steps, until convergence. In the first step, the E-step, by virtue of the Jensen inequality, the log likelihood of the incomplete data is equivalently substituted with the marginal over the hidden class labels of the log joint distribution of complete data. This expectation is computed conditioned on the observed data *S* and the current estimates of the parameters Θ. In the M-step, the expectation is maximized to obtain an update of the parameters.

In this specific application, the E-step, given the current parameters Θ^*itr*^, returns an estimate of the posterior distribution of Eq 2, in the following form

$$\begin{array}{c} p{\left(c_{j}|i_{p}\right)}^{itr}=\frac{\alpha_{j}^{itr}p{\left(i_{p}|c_{j}\right)}^{itr}}{\sum_{l=1}^{L}\alpha_{l}^{itr}p{\left(i_{p}|c_{l}\right)}^{itr}} \end{array}$$

where *p*(*i*_*p*_|*c*_*j*_)^*itr*^ is the *j*−*th* multivariate Gaussian distribution with parameters $\theta_{j}^{itr}$ computed for *i*_*p*_, given as

$$\begin{array}{c} \begin{array}{c} p{\left(i_{p}|c_{j}\right)}^{itr}=\\ \frac{1}{\sqrt{\left(2\pi \right)det\left(\Lambda_{j}^{itr}\right)}}\text{exp}(-\frac{1}{2}{\left(i_{p}-\mu_{j}^{itr}\right)}^{T}{\left(\Lambda_{j}^{itr}\right)}^{-1}\left(i_{p}-\mu_{j}^{itr}\right)) \end{array} \end{array}$$

In the M-step, based on the estimated *p*(*c*_*j*_|*i*_*p*_)^*itr*^, the parameters are updated as follows

$$\begin{array}{c} \begin{array}{c} \alpha_{j}^{itr+1}=\frac{1}{P}\sum_{p=1}^{P}p{\left(c_{j}|i_{p}\right)}^{itr}\\ \mu_{j}^{itr+1}=\frac{\sum_{p=1}^{P}i_{p}p{\left(c_{j}|i_{p}\right)}^{itr}}{\sum_{p=1}^{P}p{\left(c_{j}|i_{P}\right)}^{itr}}\\ \Lambda_{j}^{itr+1}=\frac{\sum_{p=1}^{P}p{\left(c_{j}|i_{p}\right)}^{itr}\left(i_{p}-\mu_{j}^{itr+1}\right){\left(i_{p}-\mu_{j}^{itr+1}\right)}^{T}}{\sum_{p=1}^{P}p{\left(c_{j}|i_{p}\right)}^{itr}} \end{array} \end{array}$$

The stopping criterion for the EM algorithm is set to either a maximum number of iterations or to the difference between two successive iterations, i.e., ||*θ*^*itr*+1^−*θ*^*itr*^|| ≤ *τ*, where *τ* is a small constant.

EM based methods require the initialization of the parameters. In many cases, a random initialization is suggested but, depending on data, highly time consuming. in the proposed method, we used the *K*−*means*+ + algorithm [34] to estimate the initial values of the GMM parameters *θ*. The number of clusters *L* is set in the start and optimally adjusted according to the pixel density, i.e., clusters with small number of pixels are merged with the nearest cluster. For large size images, an optimized version of the EM algorithm presented in [35] is used to speed up the clustering process.

After optimizing the parameters of the GMM and determining the posterior probabilities *p*(*c*|*i*) of Eq (2), the maximizers of such probabilities are used to assign a label *c*_*l*_, *l* ∈ [1, 2, …, *L*] to each pixel *i*_*p*_ in the RGB image *I*.

With successful classification, the user can decide what class of pixels (clean) to keep unaltered in the original RGB image *I*, and what class of pixels (degradation) to discard, i.e. to inpaint with the background class.

### Gaussian texture inpainting

After classification, the pixels belonging to the degradation class are removed and replaced by a befitting value simulating the background. The degraded pixels are treated as missing or corrupted image regions, and replaced with pertinent values that are consistent with the known non-corrupted surrounding regions. In traditional ancient document restoration methods, the noisy pixels are either replaced by a constant value or a random pixel value is assigned from the neighborhood, like in [8]. Unfortunately, in case of ancient documents with textured background, these generic fill-in creates visible artifacts and garbles the original aesthetic look of the document.

Keeping this in mind, we perform texture inpainting using Gaussian conditional simulation [31], inferring the surrounding context to preserve the natural appearance of the document. This Gaussian conditional inpainting is especially efficient to inpaint textured content, as is the case in many ancient document backgrounds. The main idea here is to fill the missing region with a conditional sample of a Gaussian model. For our application, a Gaussian texture model is estimated using an exemplar background mask. This exemplar mask is obtained randomly from the background class, as extracted in the previous section. The Gaussian model is then conditionally sampled to gradually replace the degraded pixels.

**Fig 1:**
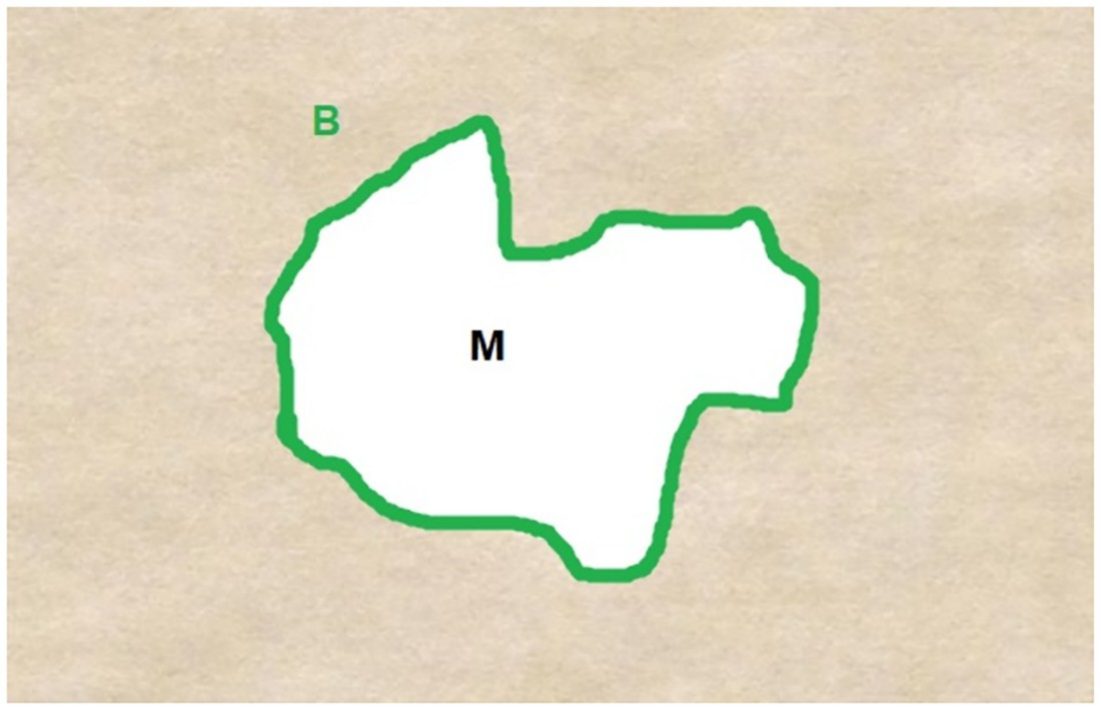
Gaussian conditional inpainting: Estimating the missing pixels value in *M* from the conditioning pixels in the set *B* located at the border.

Let $I\in R^{2}$ is the input image to inpaint and *M* ∈ *I* represents the indices of missing pixels, i.e., pixels values of *I* are known except the missing region *M*. Let *ζ* represent the outer border of the missing region *M*, with width *w* pixels as shown in Fig 1. The goal here is to estimate the pixel values in *M*, given a conditioning set *ζ*. The Gaussian model is then conditionally sampled to gradually replace the labelled bleed-through pixels, using the available neighboring pixels on the border. The conditional sample is obtained by combining an innovation component derived from an independent realization of the Gaussian model and a kriging component derived from the conditioning values [31]. The latter extends the long-range correlations and the former adds texture details, in a way that preserves the global covariance of the model. A detailed description of the Gaussian conditional inpainting can be found in [31]. Although designed to model microtextures, this algorithm is able to fill both small and large holes, whatever the regularity of the boundary. On the other hand, typical document backgrounds, representing the motif of the paper fiber, seem to fit well a homogeneous microtexture model.

## Experimental results

A set of experimental results on color ancient document images are presented to evaluate the performance of the proposed method. We compare our results with a recently published bleed-through removal method presented in [8], which is one of the most impairing degradations in ancient manuscripts. For this comparison, we use images from the well-known database of ancient documents presented in [36] and available online. This database contains 25 pairs of recto-verso images of ancient manuscripts affected by different levels of bleed-through, along with manually created ground truth binary images of the foreground text. It is worth mentioning that, while this database mainly focuses on bleed-through effects, our method can be used to remove also other document degradations, such as stains, folding marks, etc.

For the proposed method, the transformation from RGB to CIELab and CIELuv color spaces is performed using MATLAB built-in functions. We used the first 3 principal components after PCA reduction. The clustering algorithm is initiated with four classes, *L* = 4, to account for up to four different colors in the manuscript. The GMM is initialized by using the K-means function available in MATLAB. We use five iterations for the EM algorithm to find the clusters, and the Gaussian texture inpainting is initiated with pixels from the background class. The presented visual results for the method in [8] are provided by the authors.

Quantitative evaluation of ancient document restoration is a non trivial task. Generally, the efficacy is evaluated qualitatively, as in most cases the original clean image is not available.

**Fig 2:**
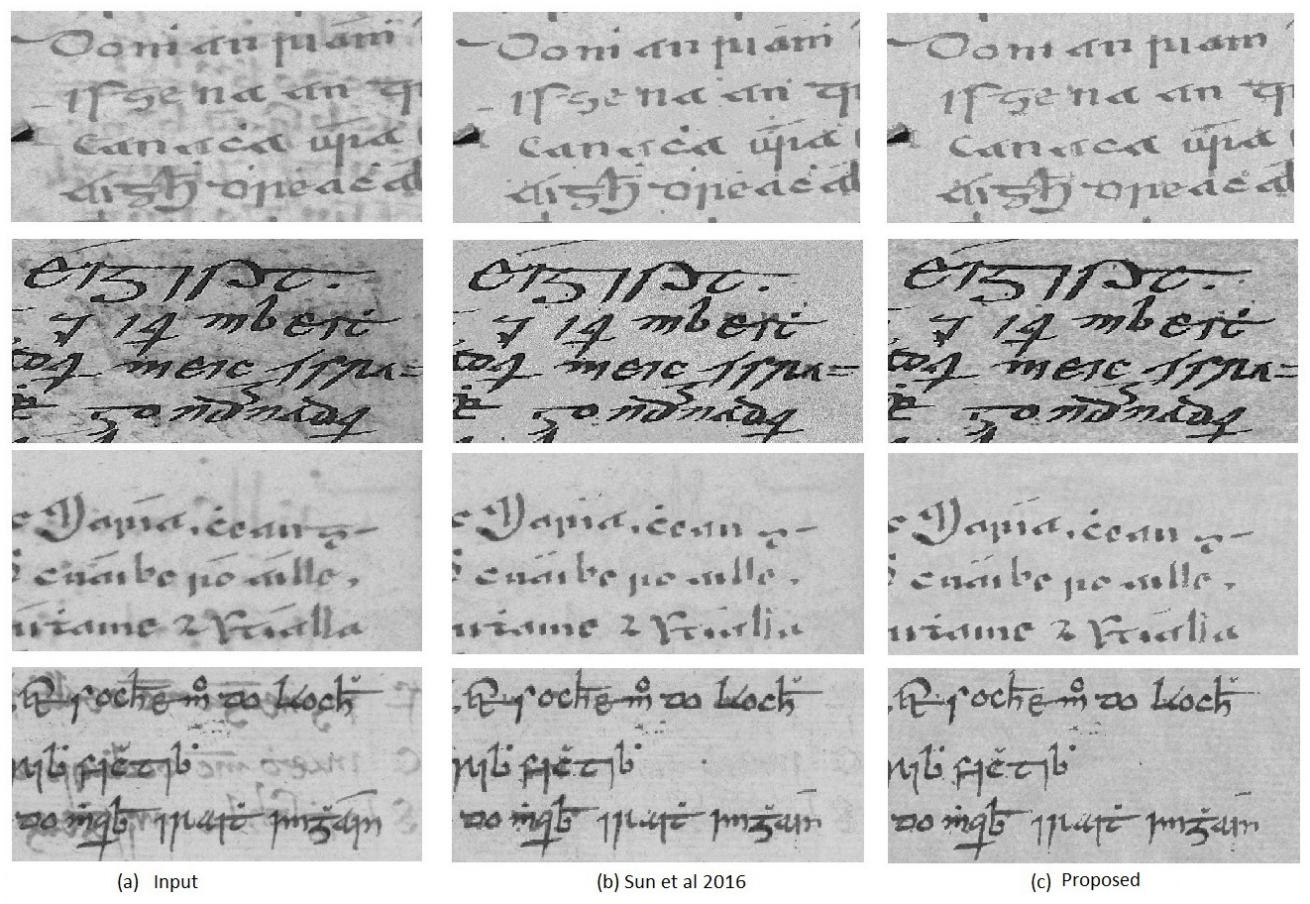
Visual comparison of bleed-through removal: (a) input degraded image, (b) restored image using [8], and (c) restored image using the proposed method.

Fig 2 shows a visual comparison of images restored by [8] and the proposed method. Note that, although our results are in color, we show them in grayscale for a fair comparison with the grayscale results of [8]. Since we are interested in preserving the original look of the document, the efficiency of the inpainting reproducing the paper texture in the background has to be evaluated as an index of the restoration quality. As can be seen, the proposed method (Fig 2(c)) produces comparatively better results in this respect. It efficiently removes the bleed-through degradation, leaving intact the foreground text, and reproducing well the background texture. The method proposed in [8] (Fig 2(b)) produces comparative results, but some strokes of the foreground text are missing and some bleed-through strokes are still visible. They also replaced the removed bleed-through pixels with a random pixel which destroy the background texture.

**Fig 3:**
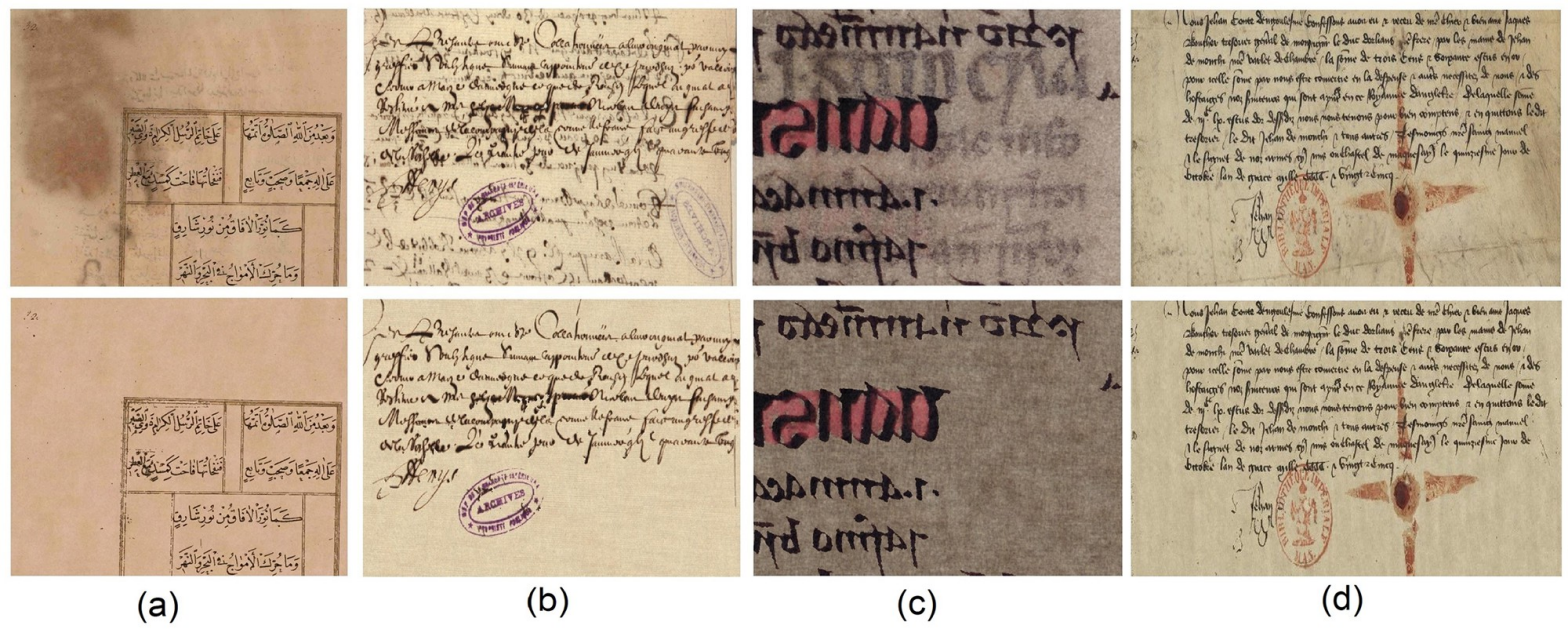
Visual examples of color document images: Input degraded image (top row), restored image using the proposed method (bottom row).

A set of clean color ancient documents, obtained by using the proposed method, is shown in Fig 3. The top row shows the input images and the bottom row displays the correspondent restored images. The image Fig 3(a) is taken from the ancient Arabic documents of the RASM, publicly available on https://www.primaresearch.org/RASM2018/. One can notice the removal of unwanted degradation in the top left side of Fig 3(a), along with the texture restoration of original document. The images Fig 3(b)–3(d) are obtained from the International Document Image Binarization Contest (DIBCO) 2017 dataset, publicly available at [1]. It is worth to note that in the second and forth columns of Fig 3, the EM algorithm was able to reduce the number of clusters from four to three, according to the number of different patterns in the images. For the first and third column, the use of four classes helped in discriminating the four different information layers of foreground text, background, degradation, and the annotations (stamps).

**Fig 4:**
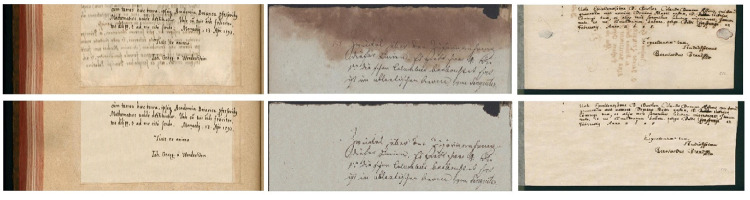
Example of images from DIBCO-2019 [37]: Input degraded image (top row), restored image using the proposed method (bottom row).

An example of restored color images from the DIBCO-2019 dataset, publicly available at [37] is presented in Fig 4. It is interesting to note how the proposed method cope with different kind of document degradation, including bleed-through, stain and see-through. In addition, the background texture in all different cases are preserved to restored the aesthetic look of the document.

### Computational complexity

Computational complexity is an important aspect to reflect the amount of time and memory required for an algorithm to execute as function of input data size. The most common way to represent the computational complexity of an algorithm is the big O notation. This provides an upper bound on the worst-case running time of an algorithm and estimate the complexity in terms of the input data size. The dominant and most repeatedly used operations in an algorithm are used to calculate the running time. The computation time of the proposed algorithm mainly depends on the repetitions of EM algorithm for the GMM parameters estimation and the Gaussian texture inapianting step. Generally, the computational complexity of EM algorithm for GMM parameter estimation is considered as $O(TK^{2n})$, where *T* is the number of iterations, *K* is the mixture components, and *n* represent number of data points. For color images, as in our case, the computational complexity will be $O(TK^{2nD})$, where *D* is the data dimension. As discussed in the experimental section, we used four classes (*K* = 4) and five iterations (*T* = 5) of EM algorithm to find the clusters. We used color images and their transformed version CIELab and CIELuv, resulting in a hybrid color space with eight distinct color features (*D* = 8). The total computational complexity of the proposed algorithm is $O(5\times 4^{16n})$, for a *n* pixels image.

In practice, on a normal desktop computer (core *i*5, 4 GB RAM, Windows 10), the proposed algorithm will take around 1.4 to 2 minutes for a color image of size 256 × 256 and 512 × 512, using the MATLAB-2018 implementation.

## Conclusion

This paper presents a degradation removal method for color ancient document images. A feature space is created using the spectral information from different color space representations of the input color image. The PCA is used to obtained an uncorrelated reduced feature space that is then passed to a GMM based classification method, along with the pixel spatial information. The pixels are classified into foreground, background, and noise, i.e. the unwanted, interfering degradation. A texture inpainting method based on Gaussian conditional simulation is then used to replace the detected degraded pixels. A set of color manuscripts, effected by different kinds of degradation, is presented to validate the proposed method. The method can be generalized to perform a selective extraction of the different layers of information usually present in the documents (stamps, pencil annotations, decorations, etc.), in order to facilitate page layout analysis. Future studies will regard the robustness of our multiple color space representation against a higher number of patterns present in the manuscripts.

## References

[1] I. Pratikakis, K. Zagoris, G. Barlas, B. Gatos, ICDAR2017 Competition on Document Image Binarization (DIBCO 2017), 14th IAPR Int. Conf. on Document Analysis and Recognition (ICDAR 2017), Proceedings, 2017.

[2] Pai Y.T. and Chang Y.F. and Ruan S.J., Adaptive thresholding algorithm: Efficient computation technique based on intelligent block detection for degraded document images, Pattern Recognition, 43, 3177–3187, 2010. doi: 10.1016/j.patcog.2010.03.014

[3] F. Westphal and N. Lavesson and H. Grahn, Document image binarization using recurrent neural networks, IAPR Int. Workshop on Document Analysis Systems (DAS2018), Proceedings, 263–268, 2018.

[4] R.C. Tensmeyer and T. Martinez, Document image binarization with fully convolutional neural networks, IAPR Int. Conf. on Document Analysis and Recognition (ICDAR 2017), Proceedings, 99–104, 2017.

[5] Q.N. Vo and S.H. Kim and H.J. Yang and G. Lee, Binarization of degraded document images based on hierarchical deep supervised network, Pattern Recognition, 568–586, 2018.

[6] Lu D. and Huang X. and Sui L., Binarization of degraded document images based on contrast enhancement, International Journal on Document. Analysis and Recognition, 21, 123–135, 2018.

[7] F. Drira and F. Le Bourgeois and H. Emptoz, Restoring Ink Bleed-Through Degraded Document Images Using a Recursive Unsupervised Classification Technique, Document Analysis Systems VII, Lecture Notes in Computer Science, 3872. Springer, 2006.

[8] B. Sun and S. Li and X-P. Zhang and J. Sun, Blind Bleed-Through Removal for Scanned Historical Document Image With Conditional Random Fields, IEEE Trans. Image Process, 5702–5712, 2016. 10.1109/TIP.2016.2614133 28114067

[9] R. Rowley-Brooke and F. Pitié and A. C. Kokaram, A Non-parametric Framework for Document Bleed-through Removal, Proc. CVPR, 2954–2960, 2013.

[10] Huang Y. and Brown M. S. and Xu D., User Assisted Ink-Bleed Reduction, IEEE Transactions on Image Processing, 19, 2646–2658, 2010. doi: 10.1109/TIP.2010.2048971 20421185

[11] Tonazzini A., Bedini L., Restoration of recto-verso colour documents using correlated component analysis, EURASIP Journal on Advances in Signal Processing, 58, 2013.

[12] Hanif M. and Tonazzini A. and Savino P. and Salerno E., Non-Local Sparse Image Inpainting for Document Bleed-Through Removal, Journal of Imaging, 4, 68–75, 2018. doi: 10.3390/jimaging4050068

[13] Tonazzini A. and Savino P. and Salerno E., A non-stationary density model to separate overlapped texts in degraded documents, Signal, Image and Video Processing, 9, 155–164, 2015. doi: 10.1007/s11760-014-0735-3

[14] Savino P., Tonazzini A., Digital restoration of ancient color manuscripts from geometrically misaligned recto-verso pairs, Journal of Cultural Heritage, 19, 511–521, 2016. doi: 10.1016/j.culher.2015.11.005

[15] J. Wang and C. L. Tan, Non-rigid registration and restoration of double-sided historical manuscripts, Int. Conf. on Document Analysis and Recognition (ICDAR), 1374–1378, 2011.

[16] R. Rowley-Brooke and F. Pitié and A. C. Kokaram, Non-rigid recto-verso registration using page outline structure and content preserving warps, Int. Workshop on Historical Document Imaging and Processing, 8–13, 2013.

[17] Savino P. and Tonazzini A. and Bedini L., Bleed-through cancellation in non-rigidly misaligned recto-verso archival manuscripts based on local registration, Int J. on Document Analysis and Recognition, 22, 163–176, 2019. doi: 10.1007/s10032-019-00323-2

[18] Cheng H.D. and Jiang X.H. and Sun Y. and Xan J., Color image segmentation: advances and prospects, Pattern Recognition, 34, 2259–2281, 2001. doi: 10.1016/S0031-3203(00)00149-7

[19] Alata O. and Quintard L., Is there a best color space for color image characterization or representation based on multivariate Gaussian mixture model?, Computer Vision and Image Understanding, 113, 867–877, 2009. doi: 10.1016/j.cviu.2009.03.001

[20] Busin L. and Vandenbroucke N. and Macaire L., Color spaces and image segmentation, Advances in Imaging and Electron Physics, 151, 65–68, 2008.

[21] Jurio A. and Pagola M. and Galar M. and Lopez-Molina C. and Paternain D., A comparison study of different color spaces in clustering based image segmentation, Information Processing and Management of Uncertainty in Knowledge-Based Systems, 81, 532–541, 2010.

[22] Chaves-González J. M. and Vega-Rodríguez M. A. and Gómez-Pulido J. A. and Sánchez-Pérez J. M., Detecting skin in face recognition systems: a colour spaces study, Digital Signal Processing, 20, 806–823, 2010. doi: 10.1016/j.dsp.2009.10.008

[23] Ruiz-Ruiz G. and Gómez-Gil J. and Navas Gracia L. M., Testing different color spaces based on hue for the environmentally adaptive segmentation algorithm EASA), Computers and Electronics in Agriculture, 68, 88–96, 2009. doi: 10.1016/j.compag.2009.04.009

[24] Orchard M.T. and Bouman C.A., Color quantization of images, IEEE Trans. on Signal Processing, 39, 2677–2690, 1991. doi: 10.1109/78.107417

[25] S. N. Gowda and Chun Yuan, ColorNet: Investigating the importance of color spaces for image classification, Asian Conference on Computer Vision, 11, 2018.

[26] Sfikas G. and Nikou C. and Galatsanos N. and Heinrich C., Spatially varying mixtures incorporating line processes for image segmentation, Journal of Mathematical Imaging and Vision, 36, 91–110, 2010. doi: 10.1007/s10851-009-0174-x

[27] Bishop C.M., Pattern Recognition and Machine Learning, New York:Springer-Verlag, 2006.

[28] Permuter H. and Francos J. and Jermyn I., A study of Gaussian mixture models of color and texture features for image classification and segmentation, Pattern Recognition, 39, 695–706, 2006. doi: 10.1016/j.patcog.2005.10.028

[29] Nikou C. and Likas C. and Galatsanos P., A Bayesian Framework for Image Segmentation With Spatially Varying Mixtures, IEEE Trans. on Image Processing, 19, 2278–2289, 2010. doi: 10.1109/TIP.2010.2047903 20378472

[30] D. Chris and H. Xiaofeng, K-means Clustering via Principal Component Analysis, Int. Conf. Machine Learning (ICML 2004), Proceedings, 2004.

[31] Galerne B. and Leclaire A., Texture Inpainting Using Efficient Gaussian Conditional Simulation, SIAM Journal on Imaging Sciences, 10, 1446–1474, 2017. doi: 10.1137/16M1109047

[32] DIBCO-2018, H-DIBCO 2018 Dataset and Evaluation Tool, http://vc.ee.duth.gr/h-dibco2018/benchmark/, Accessed on: 05 July, 2019.

[33] Blekas K. and Likas A. and Galatsanos N. and Lagaris I., A spatially constrained mixture model for image segmentation, IEEE Transaction on Neural Networks, 16, 494–498, 2005. doi: 10.1109/TNN.2004.841773 15787156

[34] D. Arthur and S. Vassilvitskii, K-Means++: The advantages of careful seeding, Proc. Symp. Discrete Algorithms, 1027–1035, 2007.

[35] Cappe O. and Moulines E., On-Line Expectation-Maximization Algorithm for Latent Data Models, Journal of the Royal Statistical Society, 71, 593–613, 2009. doi: 10.1111/j.1467-9868.2009.00698.x

[36] Rowley-Brooke R. and Pitié F. and Kokaram A. C., A ground truth bleed-through document image database. www.isos.dias.ie, Theory and Practice of Digital Libraries, 7489, 185–196, 2012. doi: 10.1007/978-3-642-33290-6_21

[37] I. Pratikakis, K. Zagoris, X. Karagiannis, L. Tsochatzidis, T. Mondal and I. Marthot-Santaniello, ICDAR 2019 Competition on Document Image Binarization (DIBCO 2019), 2019 International Conference on Document Analysis and Recognition (ICDAR), 2019 https://vc.ee.duth.gr/dibco2019/benchmark/, Accessed on: 05 July, 2019.

